# Fruits, vegetables, and bladder cancer risk: a systematic review and meta-analysis

**DOI:** 10.1002/cam4.327

**Published:** 2014-12-02

**Authors:** Ana R Vieira, Snieguole Vingeliene, Doris S M Chan, Dagfinn Aune, Leila Abar, Deborah Navarro Rosenblatt, Darren C Greenwood, Teresa Norat

**Affiliations:** 1 Department of Epidemiology and Biostatistics, School of Public Health, Imperial College LondonLondon, United Kingdom; 2 Department of Public Health and General Practice, Faculty of Medicine, Norwegian University of Science and TechnologyTrondheim, Norway; 3 Division of Biostatistics, University of LeedsLeeds, United Kingdom

**Keywords:** Bladder cancer, fruits, meta-analysis, systematic review, vegetables

## Abstract

Smoking is estimated to cause about half of all bladder cancer cases. Case–control studies have provided evidence of an inverse association between fruit and vegetable intake and bladder cancer risk. As part of the World Cancer Research/American Institute for Cancer Research Continuous Update Project, we conducted a systematic review and meta-analysis of prospective studies to assess the dose–response relationship between fruit and vegetables and incidence and mortality of bladder cancer. We searched PubMed up to December 2013 for relevant prospective studies. We conducted highest compared with lowest meta-analyses and dose–response meta-analyses using random effects models to estimate summary relative risks (RRs) and 95% confidence intervals (CIs), and used restricted cubic splines to examine possible nonlinear associations. Fifteen prospective studies were included in the review. The summary RR for an increase of 1 serving/day (80 g) were 0.97 (95% CI: 0.95–0.99) *I*^2^ = 0%, eight studies for fruits and vegetables, 0.97 (95% CI: 0.94–1.00, *I*^2^ = 10%, 10 studies) for vegetables and 0.98 (95% CI: 0.96–1.00, *I*^2^ = 0%, 12 studies) for fruits. Results were similar in men and women and in current, former and nonsmokers. Amongst fruits and vegetables subgroups, for citrus fruits the summary RR for the highest compared with the lowest intake was 0.87 (95% CI: 0.76–0.99, *I*^2^ = 0%, eight studies) and for cruciferous vegetables there was evidence of a nonlinear relationship (*P* = 0.001). The current evidence from cohort studies is not consistent with a role for fruits and vegetables in preventing bladder cancer.

## Introduction

Bladder cancer is the 11th most common cancer in the world. Age-standardized rates (per 100,000 persons per year) are higher in men than in women for both incidence (8.9 vs. 2.2) and mortality (3.3 vs. 0.9). The highest incidence rates are in Europe, the United States, and Egypt, and the lowest rates are found in sub-Saharan Africa, Asia, and South America 1. The geographic variation in incidence rates is thought to be explained by differences in the prevalence of risk factors across countries. Tobacco use, a well-established risk factor of bladder cancer is estimated to account for about half of all bladder cancer cases in both men and women and may explain the high incidence of bladder cancer in Europe and North America 2. The high incidence of bladder cancer in Egypt and other North African countries has been largely attributed to infection with *schistosoma* parasite. After the successful control of schistosomiasis, Egypt experienced a decrease in the proportion of squamous cell carcinoma cases, which are associated with schistosomiasis, but an increase in the proportion of transitional cell carcinoma that is related to smoking 3. Occupational exposure to carcinogenic aromatic amines, polycyclic aromatic hydrocarbons, and chlorinated hydrocarbons is a risk factor of urothelial bladder cancer and increasing evidence supports an influence of genetic predisposition 4.

The role of dietary factors in the development of bladder cancer has also been investigated, but the evidence is not clear. In the World Cancer Research/American Institute for Cancer Research (WCRF/AICR) Second Expert Report from 2007 it was concluded that the evidence of a relationship between incidence of bladder cancer and milk intake (decreased risk) and arsenic from drinking water (increased risk) was “limited but suggestive” and that the evidence of other nutritional factors was too limited for a conclusion to be drawn 5.

Amongst dietary factors, fruits and vegetables have been investigated because they provide an abundant source of nutrients and phytochemicals with potentially anticarcinogenic properties. Since the 2007 WCRF/AICR report, seven cohort studies from six publications have published on this association 6–11. As part of the WCRF/AICR Continuous Update Project, we conducted a systematic review and meta-analysis of cohort studies to assess the relationship of fruits and vegetables intake and bladder cancer risk. We investigated the strength and shape of the relationship by performing linear and nonlinear dose–response meta-analyses. We investigated the total intake of fruits and vegetables and also subgroups of vegetables for which there was enough information to conduct meta-analyses.

## Methods

### Search strategy

The PubMed database was searched up to December 2013 for studies of fruit and vegetables and bladder cancer risk. The protocol followed for the review can be found at: http://www.dietandcancerreport.org/cancer_resource_center/downloads/cu/CUP_bladder_cancer_protocol.pdf and includes the specific search criteria used. Furthermore, the reference list of the included articles and published meta-analyses and reviews was searched.

### Study selection

The inclusion criteria were (1) being a randomized trial, cohort study, case-cohort study or nested case–control study; (2) report the estimates of the relative risk (RR) (e.g., hazard ratio [HR], risk ratio or odds ratio) and 95% confidence intervals (CIs) for the association of fruit and/or vegetables and bladder cancer or urothelial cancer incidence or mortality; (3) provide a quantitative measure of the intake to be used in the dose–response analysis. When the same study published more than one article on fruit and vegetables and bladder cancer, we selected the newest publication with the largest number of cases.

**Figure 1:**
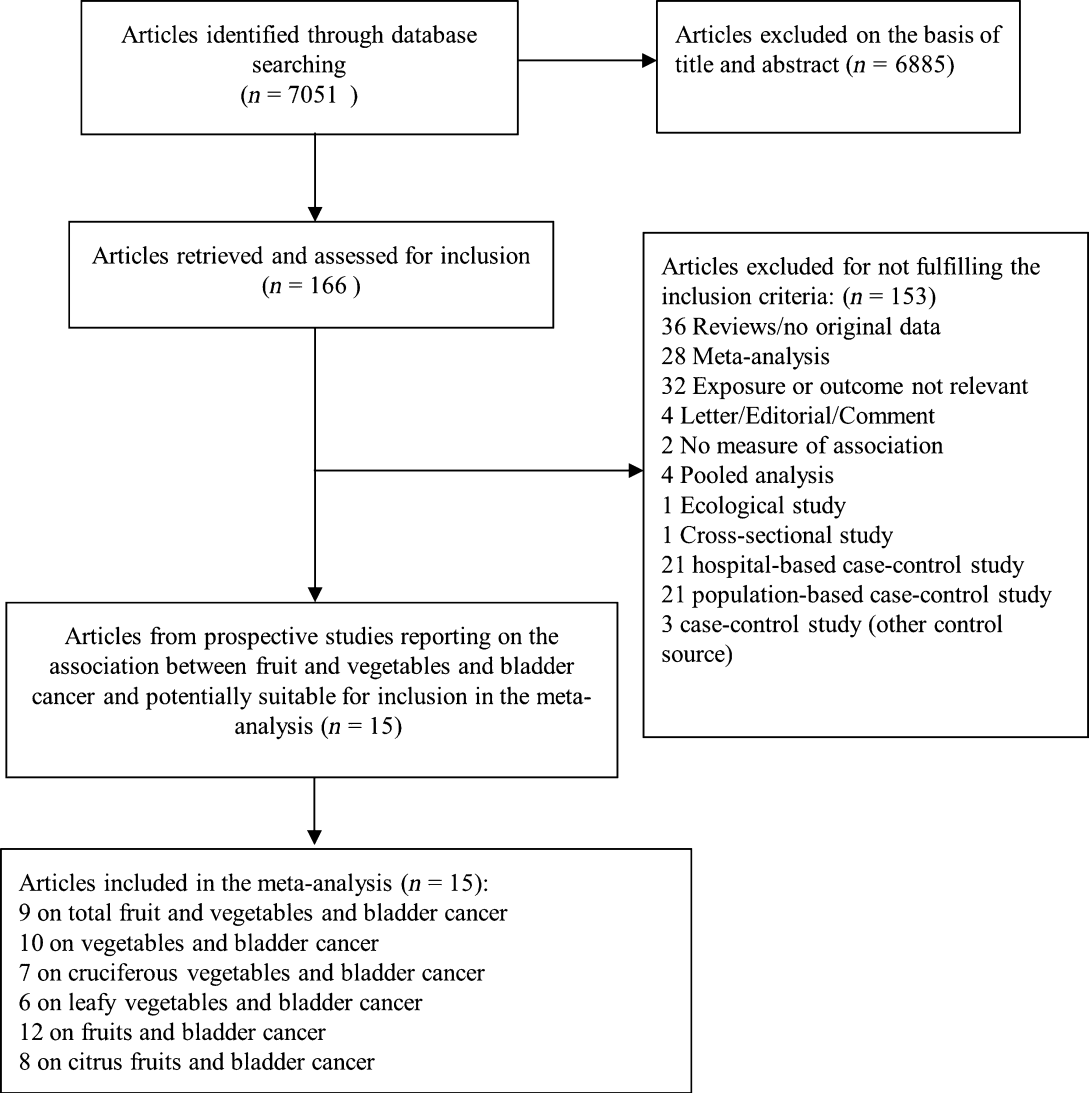
Flowchart of study selection.

From 7051 articles identified, 6885 articles were excluded based on the abstract and title and 166 articles were retrieved and assessed for potential inclusion. Of these, 153 articles which did not meet the inclusion criteria were excluded and 15 articles (15 studies) which met the inclusion criteria were included (Flowchart of study selection—Fig.1).

### Data extraction

The data extracted for each article were: first author's last name, publication year, country where the study was conducted, the study name, follow-up period, sample size, gender, age, number of cases, dietary assessment method (type, number of food items, and whether it had been validated), type of fruit and/or vegetable, amount of intake, RRs and 95% CIs and adjustment variables. The search and data extraction of articles published up to June 2006 was conducted by several reviewers at the University of Bristol during the systematic literature review for the WCRF/AICR Second Expert Report (available online: http://www.dietandcancerreport.org/cancer_resource_center/downloads/SLR/Bladder_SLR.pdf). The search and extraction from June 2006 and up to December 2013 was conducted by the CUP team at Imperial College London.

### Statistical methods

We calculated summary RRs and 95% CIs for the highest compared to the lowest levels of fruits and vegetables intake using random effect models to account for anticipated heterogeneity. The natural logarithm of the RRs was weighted by the method of Dersimonian and Laird and then pooled across studies 12. The method described by Greenland and Longnecker 13,14 was used to estimate linear trends and 95% CIs from the natural logs of the RR and respective CI across categories of fruit and vegetable intake. In order to use this method, at least three categories of intake and the number of cases and person-years or noncases per category was required. When studies reported only the total number of cases or total person-years and the exposure was defined in quantiles, the distribution of cases or person-years was calculated dividing the total number by the number of quantiles. Whenever reported, the mean or median intake by category was assigned to the corresponding RR. The midpoint was calculated for studies that only reported a range of intake by category. When the intake range was open-ended we assumed that its width was the same as the adjacent category. For studies presenting the exposure per given unit of energy intake, we rescaled it using the mean energy intake provided. We expressed the dose–response by increments of 1 serving/day for fruits and vegetables and 1 serving/week for cruciferous and leafy vegetables, because most of the studies reported the intake in servings. For studies that reported in grams, the conversion unit of 80 g as a serving size was used, for comparison with other meta-analyses of fruit and vegetable intake and cancer risk 15.Where results were only presented separately for men and women, they were combined using a fixed effects meta-analysis before being pooled with other studies to ensure that between-study heterogeneity was not underestimated. Between-study heterogeneity was assessed using Cochran *Q* test and the percentage of total variation in study estimates attributable to between-study heterogeneity (*I*^*2*^). Heterogeneity was explored by stratified analysis by sex, geographic location and smoking status (when the studies provided sufficient data), and by visual inspection of the forest plots. Most of the studies adjusted the analysis for smoking status. Potential small-study effects, such as publication bias, were explored using Egger's test and funnel plots.

To examine possible nonlinear associations, we calculated restricted cubic splines for each study with more than three categories of exposure, using three fixed knots at 10%, 50%, and 90% through the total distribution of the reported intake, and combined them using multivariate meta-analysis 16,17. Only studies which presented more than three categories could be included in the nonlinear analysis. A two-tailed *P* < 0.05 was considered statistically significant. All the studies included provided adjusted results.

Stata version 12 software (StataCorp, College Station, TX) was used for the statistical analyses.

## Results

Fifteen cohort studies (*n*) from 15 publications were included in the analyses 6–11,18–27. Nine studies were on total fruit and vegetables, 10 studies on vegetables, seven studies on cruciferous vegetables, six studies on leafy vegetables, 12 studies on fruits, and eight studies on citrus fruits. The outcome was urothelial cancer in four studies and bladder cancer in 10 studies. Six studies were from the U.S.A., five studies were European, and four studies were from Asia. Eleven studies were on men and women, three studies were on men, and one study was on women (Table S1). A summary of the results of the meta-analyses is provided in Table1.

**Table 1:** Summary table of results.

Exposures	Total fruit and vegetables	Vegetables	Cruciferous vegetables	Leafy vegetables	Fruits	Citrus fruits
Highest versus lowest analysis						
*n*/*N*	9/2588	10/5119	7/2437	6/2310	12/5329	8/2293
HvL RR (95% CI)	0.89 (0.75–1.05)	0.92 (0.84–1.01)	0.85 (0.69–1.06)	0.90 (0.78–1.04)	0.91 (0.82–1.00)	0.87 (0.76–0.99)
*I*^2^, *P*_heterogeneity_	*I*^2^ = 34%, *P*_h_ = 0.16	*I*^2^ = 5%, *P*_h_ = 0.39	*I*^2^ = 63%, *P*_h_ = 0.02	*I*^2^ = 0%, *P*_h_ = 0.69	*I*^2^=11%, *P*_h_ = 0.34	*I*^2^ = 0%, *P*_h_ = 0.88
Stratified highest versus lowest analysis by smoking status						
Never smokers (*n*)	5	3	1		3	
HvL RR (95% CI)	0.85 (0.62–1.18)	0.95 (0.68–1.33)	0.26 (0.10–0.65)		0.91 (0.64–1.28)	
*I*^2^, *P*_heterogeneity_	*I*^2^ = 0%, *P*_h_ = 0.42	*I*^2^ = 0%, *P*_h_ = 0.85			*I*^2^ = 0%, *P*_h_ = 0.94	
Former smokers (*n*)	5	3	1		3	
HvL RR (95% CI)	0.96 (0.77–1.19)	0.96 (0.76–1.20)	0.70 (0.43–1.15)		1.00 (0.75–1.34)	
*I*^2^, *P*_heterogeneity_	*I*^2^ = 0%, *P*_h_ = 0.73	*I*^2^ = 0%, *P*_h_ = 0.95			*I*^2^ = 38%, *P*_h_ = 0.18	
Current smokers (*n*)	5	3	1		3	
HvL RR (95% CI)	0.83 (0.64–1.09)	0.80 (0.61–1.05)	0.89 (0.36–2.17)		0.78 (0.53–1.13)	
*I*^2^, *P*_heterogeneity_	*I*^2^ = 0%, *P*_h_ = 0.74	*I*^2^ = 0%, *P*_h_ = 0.45			*I*^2^ = 44%, *P*_h_ = 0.15	
	1 serving/day	1 serving/day	1 serving/week	1 serving/week	1 serving/day	1 serving/day
Linear dose–response meta-analysis						
*n*/*N*	8/2508	10/5119	7/2437	6/2310	12/5329	8/2988
RR (95% CI)	0.97 (0.95–0.99)	0.97 (0.94–1.00)	0.98 (0.94–1.02)	0.98 (0.95–1.01)	0.98 (0.96–1.00)	0.98 (0.93–1.03)
*I*^2^ *P*_heterogeneity_	*I*^2^ = 0%, *P*_h_ = 0.76	*I*^2^ = 10%, *P*_h_ = 0.35	*I*^2^ = 58%, *P*_h_ = 0.04	*I*^2^ = 0%, *P*_h_ = 0.74	*I*^2^ = 0%, *P*_h_ = 0.51	*I*^2^ = 0%, *P*_h_ = 0.61
Stratified linear dose- response by sex						
Men (*n*)	4	5	3	1	6	2
RR (95% CI)	0.99 (0.96–1.01)	0.98 (0.93–1.02)	0.98 (0.91–1.06)	0.99 (0.93–1.05)	0.98 (0.94–1.02)	0.99 (0.92–1.07)
*I*^2^, *P*_heterogeneity_	*I*^2^ = 0%, *P*_h_ = 0.59	*I*^2^ = 20%, *P*_h_ = 0.28	*I*^2^ = 77%, *P*_h_ = 0.01		*I*^2^ = 19%, *P*_h_ = 0.29	*I*^2^ = 0%, *P*_h_ = 0.54
Women (*n*)	2	3	2		3	3
RR (95% CI)	0.93 (0.81–1.07)	0.97 (0.80–1.18)	0.97 (0.88–1.07)		0.97 (0.87–1.09)	0.87 (0.64–1.20)
*I*^2^, *P*_heterogeneity_	*I*^2^ = 87%, *P*_h_<0.01	*I*^2^ = 76%, *P*_h_ = 0.02	*I*^2^ = 68%, *P*_h_ < 0.07		*I*^2^ = 70%, *P*_h_ = 0.04	*I*^2^ = 72%, *P*_h_ = 0.03
Stratified linear dose- response by location						
USA (*n*)	4	5	3	1	6	3
RR (95% CI)	0.97 (0.94–0.99)	0.97 (0.93–1.02)	0.96 (0.92–1.01)	0.99 (0.93- 1.05)	0.98 (0.94–1.01)	0.99 (0.94–1.04)
*I*^2^ *P*_heterogeneity_	*I*^2^ = 0%, *P*_h_ = 0.86	*I*^2^ = 18%, *P*_h_ = 0.30	*I*^2^ = 53%, *P*_h_ = 0.12		*I*^2^ = 15%, *P*_h_ = 0.32	*I*^2^ = 0%, *P*_h_ = 0.48
Europe (*n*)	4	5	4	4	4	2
RR (95% CI)	0.99 (0.95–1.03)	0.97 (0.92–1.03)	1.00 (0.92–1.09)	0.97 (0.94–1.01)	0.99 (0.95–1.03)	0.95 (0.83–1.09)
*I*^2^, *P*_heterogeneity_	*I*^2^ = 0%, *P*_h_ = 0.39	*I*^2^ = 25%, *P*_h_ = 0.26	*I*^2^ = 69%, *P*_h_ = 0.04	*I*^2^ = 0%, *P*_h_ = 0.50	*I*^2^ = 0%, *P*_h_ = 0.45	*I*^2^ = 0%, *P*_h_ = 0.83
Asia (*n*)				1	1	2
RR (95% CI)				0.94 (0.82–1.07)	0.77 (0.42–1.42)	0.50 (0.19–1.31)
*I*^2^, *P*_heterogeneity_						*I*^2^ = 0%, *P*_h_ = 0.37
Nonlinear dose–response analysis						
*n*/*N*	7/2437	8/4101	7/2437	4/1275	8/4101	5/1844
*P*_non-linearity_	*P* = 0.06	*P* = 0.001	*P* < 0.001	*P* = 0.29	*P* = 0.43	*P* = 0.15

*P*_h_, *P* for heterogeneity;

*n*, number of studies;

*N*, number of cases.

### Fruit and vegetables

**Figure 2:**
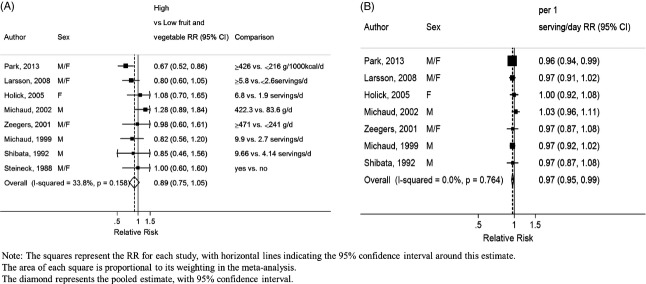
Fruit and vegetables and bladder cancer. (A) Highest compared to lowest analysis of fruit and vegetables and bladder cancer. (B) Dose–response meta-analysis of fruit and vegetables and bladder cancer.

**Figure 3:**
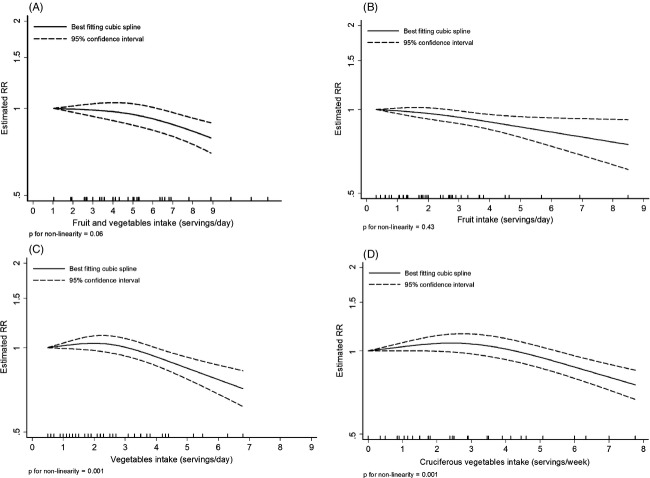
Nonlinear dose–response analysis. (A) Nonlinear analysis of fruit and vegetables intake and bladder cancer. (B) Nonlinear analysis of fruit intake and bladder cancer. (C) Nonlinear analysis of vegetables intake and bladder cancer. (D) Nonlinear analysis of cruciferous vegetables and bladder cancer.

The overall RR for the highest compared with the lowest category of fruits and vegetables intake was 0.89 (95% CI: 0.75–1.05, *I*^2^ = 34%, *P*_heterogeneity_ = 0.16) across nine studies (2588 cases [*N*]) (Fig.2A). The summary RR for an increase of 1 serving/day was 0.97 (95% CI: 0.95–0.99, *I*^2^ = 0%, *P*_heterogeneity_ = 0.76) across eight studies (2508 cases) (Fig.2B). All included studies were adjusted for smoking status. There was no significant evidence of publication bias such as small-study effects with Egger's test, *P* = 0.09 but there was some asymmetry in the funnel plot (Fig. S1) that appeared to be driven by the stronger inverse association in women observed in the Multiethnic Cohort study (MEC) 6. After stratification by sex, the RR per 1 serving/day was 0.99 (95% CI: 0.96–1.01, *I*^2^ = 0%, *P*_heterogeneity_ = 0.59, *n* = 4) for men and 0.93 (95% CI: 0.81–1.07, *I*^2^ = 87%, *P*_heterogeneity_ < 0.01, *n* = 2) for women. In all studies, the association between bladder cancer and fruits and vegetables was attenuated after adjustment for tobacco use. Five studies reported that the associations did not significantly vary across smoking status 6,9,19,20,22. In stratified analyses, there was no evidence that results differed by geographic location or smoking status (Table1). There was no significant evidence of nonlinearity (*P*_non-linearity_ = 0.06). A decrease in risk observed from intakes of more than 5 servings/day is largely driven by one cohort with higher reported intakes (Fig.3A).

### Vegetables

**Figure 4:**
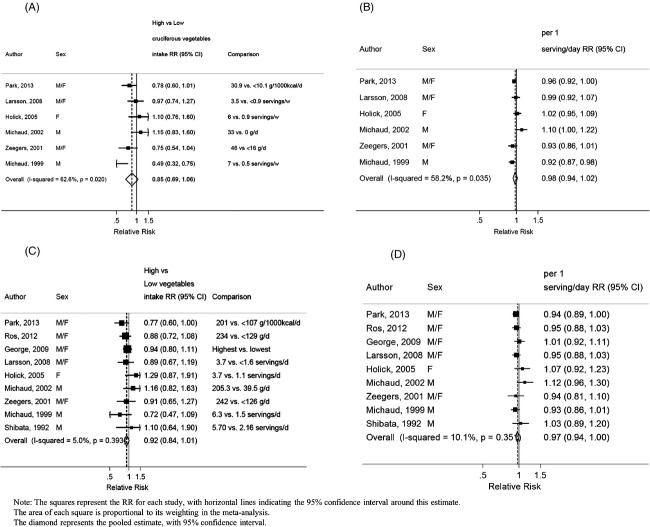
Vegetables, cruciferous vegetables and bladder cancer. (A) Highest compared to lowest analysis of cruciferous vegetables and bladder cancer. (B) Dose–response meta-analysis of cruciferous vegetables and bladder cancer risk. (C) Highest compared to lowest analysis of vegetables and bladder cancer risk. (D) Dose–response meta-analysis of vegetables and bladder cancer risk.

The overall RR for the highest compared with the lowest vegetable intake was 0.92 (95% CI: 0.84–1.01, *I*^2^ = 5%, *P*_heterogeneity_ = 0.39) across 10 studies (5119 cases) (Fig.4C).The summary RR for an increase of 1 serving/day was 0.97 (95% CI: 0.94–1.00, *I*^2^ = 10%, *P*_heterogeneity_ = 0.35) across 10 studies (5119 cases) (Fig.4D). All included studies were adjusted for smoking status. There was some evidence of publication bias (*P* = 0.02). The funnel plot showed that larger studies tended to report stronger inverse associations compared to smaller studies (Fig. S2). After stratification by sex, the RR per 1 serving/day was 0.98 (95% CI: 0.93–1.02, *I*^2^ = 20%, *P*_heterogeneity_ = 0.28, *n* = 5) for men and 0.97 (95% CI: 0.80–1.18, *I*^2^ = 76%, *P*_heterogeneity_ = 0.02, *n* = 3) for women. There was evidence of a nonlinear association with a decrease in bladder cancer risk from intakes of more than 4 servings/day *P*_non-linearity_ = 0.001 (Fig.3C).

Most studies reported similar results across smoking status categories 7,9,19,20,22. In the MEC study 6 the inverse association with vegetable intake was similar across smoking strata in women but in men a significant inverse trend was observed in current smokers (RR highest vs. lowest: 0.40, 95% CI: 0.18–0.90; *P*_trend_ = 0.02) but not in never or former smokers. In the EPIC study 7 there was no significant interaction with smoking, but lower risks of bladder cancer were observed in never and former smokers with higher consumption of vegetables, but not in current smokers. Overall, there was no evidence that results differed by smoking status or geographic location (Table1).

### Cruciferous vegetables

The overall RR for the highest compared with the lowest intake was 0.85 (95% CI: 0.69–1.06, *I*^2^ = 63%, *P*_heterogeneity_ = 0.02) across seven studies (2437 cases) (Fig.4A). The summary RR for an increase of 1 serving/week was 0.98 (95% CI: 0.94–1.02, *I*^2^ = 58%, *P*_heterogeneity_ = 0.04) across seven studies (2437 cases) (Fig.4B). All included studies were adjusted for smoking status.

One study found no evidence that the association between cruciferous vegetables and risk of bladder cancer was modified by smoking status (data not shown) 19. The only study that stratified the analysis by smoking status observed a strong inverse association between cruciferous vegetable intake and bladder cancer in nonsmokers (RR = 0.26; 95% CI: 0.10–0.65, ≥4.5 vs. <1.5 servings/week) and weak nonsignificant inverse associations in past (RR = 0.70; 95% CI: 0.43–1.15, ≥4.5 vs. <1.5 servings/week) and current smokers (RR = 0.89; 95% CI: 0.36–2.17, ≥4.5 vs. <1.5 servings/week) 20. There was no evidence that results differed by geographic location (Table1).

There was no evidence of publication bias (*P* = 0.50) but the number of studies was small. There was evidence of a nonlinear association with a decrease in bladder cancer risk from intakes of more than 3 servings/week, *P*_non-linearity_ = 0.001 (Fig.3D).

### Leafy vegetables

The overall RR for the highest compared with the lowest intake was 0.90 (95% CI: 0.78–1.04, *I*^2^ = 0%, *P*_heterogeneity_ = 0.69) across six studies (2310 cases) (Fig. S3). The summary RR for an increase of 1 serving/week was 0.98 (95% CI: 0.95–1.01, *I*^2^ = 0%, *P*_heterogeneity_ = 0.74) across six studies (2310 cases) (Fig. S4). There was no evidence of publication bias (*P* = 0.17), though based on few studies. There was no evidence of a nonlinear association, *P*_non-linearity_ = 0.29. The included studies did not stratify the analysis by smoking status.

### Fruits

**Figure 5:**
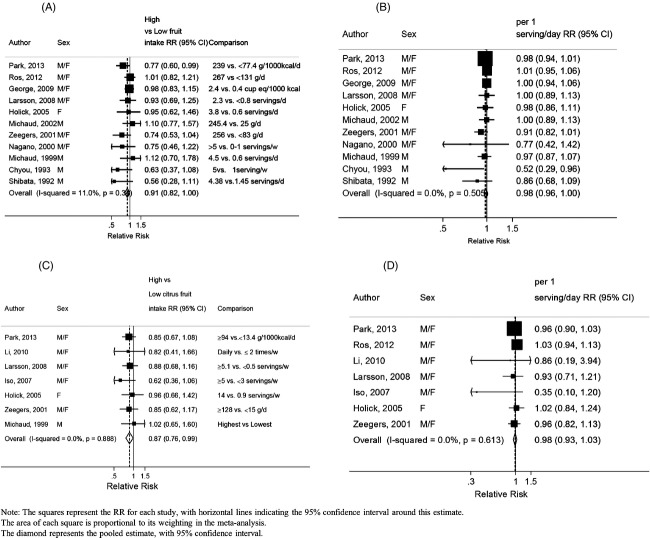
Fruits, citrus fruits, and bladder cancer. (A) Highest compared to lowest analysis of fruit and bladder cancer. (B) Dose–response meta-analysis of fruit and bladder cancer risk. (C) Highest compared to lowest analysis of citrus fruits and bladder cancer. (D) Dose–response meta-analysis of citrus fruits and bladder cancer risk.

The overall RR for the highest compared with the lowest intake was 0.91 (95% CI: 0.82–1.00, *I*^2^ = 11%, *P*_heterogeneity_ = 0.34) across 12 studies (5329 cases) (Fig.5A). The summary RR for an increase of 1 serving/day was 0.98 (95% CI: 0.96–1.00, *I*^2^ = 0%, *P*_heterogeneity_ = 0.51) across 12 studies (5329 cases) (Fig.5B). All included studies were adjusted for smoking status. There was no evidence of publication bias (*P* = 0.48). After stratification by sex, the RR per 1 serving/day was 0.98 (95% CI: 0.94–1.02, *I*^2^ = 19%, *P*_heterogeneity_ = 0.29, *n* = 6) for men and 0.97 (95% CI: 0.87–1.09, *I*^2^ = 70%, *P*_heterogeneity_ = 0.04, *n* = 3) for women. There was no evidence of a nonlinear association, *P*_non-linearity_ = 0.43.

Three studies reported the risk across smoking status categories 6,7,22. In the MEC study the nonsignificant inverse association with fruit intake was similar across smoking strata in men 6. The Netherlands Cohort Study observed similar results for men and women 22. In the EPIC study 7 inverse associations were observed among never and former smokers but not in current smokers. The multiplicative interaction test was not significant. Overall, there was no evidence that results differed by smoking status or geographic location (Table1).

### Citrus fruits

The overall RR for the highest compared with the lowest intake was 0.87 (95% CI: 0.76–0.99, *I*^2^ = 0%, *P*_heterogeneity_ = 0.88) across eight studies (2293 cases) (Fig.5C). After excluding the only study on mortality 10, the overall result was nonsignificant 0.88 (95% CI: 0.77–1.01, *I*^2^ = 0%, *P*_heterogeneity_ = 0.98). The summary RR for an increase of 1 serving/day was 0.98 (95% CI: 0.93–1.03, *I*^2^ = 0%, *P*_heterogeneity_ = 0.62) across eight studies (2988 cases) (Fig.5D). All, except one study 10, adjusted for smoking status. There was no evidence of publication bias with Egger's test, *P* = 0.38. There was no evidence of a nonlinear association, *P*_non-linearity_ = 0.15. The included studies did not stratify the analysis by smoking status. One study that found no evidence of association between citrus fruits and risk of bladder cancer reported that the relationship was not modified by smoking status 19.

## Discussion

In this meta-analysis, the consumption of total fruit and vegetables, total vegetables, and total fruit was not associated with lower risk of bladder cancer. No association was observed in the meta-analysis comparing the highest with the lowest intake of fruit and vegetables and a modest inverse association was observed in the dose–response meta-analysis. The shape of the nonlinear curves showed a trend toward a decrease in risk although this might be driven by two studies with higher reported intakes 6,22. In all studies, any observed associations were attenuated after adjustment for smoking status. There was higher heterogeneity across studies in women compared to men, mainly explained by the results of one study 6 that showed a strong significant inverse association of fruit and vegetables and bladder cancer in women, but not in men.

A significant association was observed for citrus fruits in the analysis of the highest compared with the lowest intake, however this was nonsignificant after excluding the only study with mortality as outcome. The dose–response meta-analysis was nonsignificant. Citrus fruits are rich in vitamin C and dietary vitamin C was not associated with bladder cancer risk in three cohort studies 19,28,29. No association of plasma vitamin C levels with risk of urothelial cell cancer was reported by an European prospective study 30.

Although the results of several case–control studies have reported inverse association of fruits and vegetables and bladder cancer risk, previous meta-analyses that included cohort studies and case–control studies have shown different results between the two study types. In a meta-analysis of cohort and case–control studies, lower compared with higher consumption of vegetables was not related to bladder cancer in cohort studies (RR = 1.09, 95% CI: 0.76–1.54, *n* = 3, *N* = 203) but lower vegetables consumption was associated with an increased risk in case–control studies (RR = 1.18, 95% CI: 1.01–1.31, *n* = 7, *N* = 2463). Similarly, lower compared to higher consumption of fruit was associated with an increased risk of bladder cancer in cohort and case–control studies combined (RR = 1.47, 95% CI: 1.08–2.00, *n* = 7, *N* = 2208), but the association was not significant when restricted to cohort studies (RR = 1.42, 95% CI: 0.98–2.06, *n* = 3) 31. In another meta-analysis 15, the summary RR were 0.91, 95% CI: 0.82–1.00, *P*_heterogeniety_ = 0.12, *n* = 6 and 0.81, 95% CI: 0.73–0.91, *P* < 0.01, *n* = 8 for an increase of 100 g of vegetables and fruits respectively, but only two and three cohort studies were included and when the analyses were restricted to cohort studies, the associations were slightly attenuated.

A meta-analysis of citrus fruit intake and bladder cancer risk 32 reported a significant inverse association for the highest compared with the lowest analysis in case–control studies (RR = 0.77, 95% CI: 0.64–0.92, *n* = 8, *N* = 4729) and a nonsignificant association for cohort studies (RR = 0.96, 95% CI: 0.87–1.07, *n* = 6, *N* = 2643). Comparing with our meta-analysis, Liang et al. 32 only performed highest compared with lowest analysis while we also conducted linear and nonlinear analyses. They included the EPIC study 33 in their highest compared with lowest analysis while we only included it in the dose–response meta-analysis because the study only provided HR (95% CI) for continuous increment of citrus fruits intake 7. The Netherlands Cohort Study 22 was missed by Liang et al. 32 and studies with mortality as endpoint were excluded, while we showed results including and excluding the only study on mortality 10. When our highest compared with lowest analysis was restricted to studies on bladder cancer incidence the overall result we obtained was similar to the result of Liang et al. 32.

The high antioxidant content of fruit and vegetables may reduce or prevent the oxidative damage caused by cigarette smoking. Smokers may benefit the most from fruits and vegetables, because they have been shown to have lower antioxidant levels than nonsmokers although this has not been consistently shown 34. Bladder cancer is associated with smoking 35,36. Most of the studies included in this quantitative review reported no significant interaction by smoking of the association of fruits and vegetables intake with bladder cancer. In the MEC, bladder cancer risk was inversely associated with a “vegetable dietary pattern” in current smokers but not in former smokers and nonsmokers 6 and in the EPIC study, a higher consumption of fruit and vegetables was associated with a lower risk of bladder cancer among never and former smokers but not in current smokers 7. All the studies, except one 10, included in the meta-analyses were adjusted for smoking. Five studies 6,7,9,19,22 were included in the highest compared with lowest stratified analysis by smoking status and no difference of association emerged. There were not enough data to conduct a dose–response analysis stratified by smoking status.

Our meta-analysis has several limitations. First, and although in order to conduct the meta-analysis in servings/day, we had to use a conversion unit of 80 g as equivalent to a serving size for studies which reported the consumption of fruit and vegetables in g/day 7,22,27, g/1000 kcal/day 6 or cup equivalent/1000 kcal 8. This conversion may introduce some additional error because different fruits and vegetables may have different serving sizes. However, the results were mainly consistent with the meta-analysis of the highest compared with the lowest intakes, indicating that the approximation should not have masked any existing association. All the included studies assessed the dietary intake with food frequency questionnaires (FFQ). In two studies the RR estimates were corrected for dietary measurement error 6,7 and in two studies, repeated dietary assessment were used 19,20. Only in one of these studies 6, fruits and vegetables were inversely associated with the risk of invasive bladder cancer in women and current smoker men—but not in never and former smoker men. Some studies have reported inverse associations between plasma carotenoids (as markers of fruit and vegetable intake) and urothelial cell carcinoma or bladder cancer 30,37–39, but the number of studies is limited. Only studies with more than three categories of intake could be included in the nonlinear dose–response meta-analyses using restricted cubic splines therefore the results were repeated using fractional polynomial models that allow the inclusion of more studies, but are more sensitive to extreme values. With the fractional polynomial models nonlinearity was observed for fruits and vegetables (*P*_non-linearity_ = 0.04, *n* = 8 and for cruciferous vegetables (*P*_non-linearity_ = 0.03, *n* = 7). However, the decreased risk observed at higher intake levels was driven for a small number of observations and no firm conclusion can be made. Although no small-study effects such as publication bias were identified, the total number of studies was small, so we cannot exclude bias from this source.

This meta-analysis has strengths, including the prospective design of the studies included in the analyses that avoids the potential recall bias and selection bias from case–control studies. Contrary to what has been suggested in previous case–control studies, the current evidence from cohort studies does not support that fruits and vegetables can protect against bladder cancer. However, based on the limited number of studies available, an association with specific types of fruits or vegetables, such as citrus fruits or cruciferous vegetables cannot be excluded.

## Supporting Information

**Table S1.** Table of study characteristics.

**Figure S1.** Funnel plot of fruit and vegetables and bladder cancer. **Figure S2.** Funnel plot of vegetables and bladder cancer. **Figure S3.** Highest compared to lowest analysis of leafy vegetables and bladder cancer risk. **Figure S4.** Dose–response meta-analysis of leafy vegetables and bladder cancer risk.
