# Supplementary material for: Fruits, vegetables, and bladder cancer risk: a systematic review and meta-analysis
Source: Cancer Med. 2014 Dec 2;4(1):136–46. doi: 10.1002/cam4.327 (PMC4312127; doi:10.1002/cam4.327)
Supplement: Supplementary file 1 [file cam40004-0136-sd1.doc]

Supplementary Table 1 Table of study characteristics

| **Author, year, country/**  **location** | **Study** | **Follow-up period** | **Study size, age, sex, number of cases** | **Cancer outcome** | **Dietary assessment** | **Exposure** | **Comparison (highest vs lowest)** | **RR (95%CI) (highest vs lowest)** | **Adjustment for confounders** |
| --- | --- | --- | --- | --- | --- | --- | --- | --- | --- |
| Park, 2013,  USA | Multiethnic Cohort Study | 1993/1996-2007, 12.5 years | 185885 participants (83694 M 102191 F ), age 45-75y, 581cases (429 M, 152 F) | Invasive bladder cancer | Validated Quantitative FFQ | Fruits and vegetables | ≥426 vs. <216 g/1000kcal/day | 0.67(0.52-0.86) | Age, ethnicity, energy-intake, first-degree family history of bladder cancer, employment in a high-risk industry, smoking status, average number of cigarettes, squared average number of cigarettes, number of years smoked (time-dependent), number of years since quitting (time-dependent), interactions of ethnicity with smoking status, average number of cigarettes, and squared average number of cigarettes. |
|  |  |  |  |  |  | Vegetables | ≥201 vs. <107 g/1000kcal/day | 0.77(0.60-1.00) |  |
|  |  |  |  |  |  | Cruciferous vegetables | ≥30.9 vs. <10.1 g/1000kcal/day | 0.78(0.60-1.01) |  |
|  |  |  |  |  |  | Fruits | ≥239 vs. <77.4 g/1000kcal/day | 0.77(0.60-0.99) |  |
|  |  |  |  |  |  | Citrus fruits | ≥94 vs. <13.4 g/1000kcal/day | 0.85(0.67-1.08) |  |
| Ros, 2012, Europe | European Prospective Investigation into Cancer and Nutrition | 8.9 years | 468,656 participants (40629 M 328027 F), age 25-70y, 947 cases (667 M, 280 F) | Urothelial cell carcinoma | Validated FFQ | Vegetables | ≥234 vs. <129  g/d | 0.88(0.72-1.08) | Age at entry, sex and centre and adjusted for smoking status (never, former, current), duration of smoking (former and current smokers), lifetime intensity of smoking (former and current smokers), energy intake from fat and non-fat sources. |
|  |  |  |  |  |  | Leafy vegetables | ≥30.7 vs. <7.60 g/day | 0.80(0.62-1.03) |  |
|  |  |  |  |  |  | Fruits | ≥267 vs. <131  g/d | 1.01(0.82-1.21) |  |
| Li, 2010,  Japan | Ohsaki cohort study | 1995-2003  9 years | 42470 participants  (20222M 22248 F), age 40-79y,73 cases | Bladder cancer | Validated 40-item FFQ | Citrus fruits | Daily vs. ≤ 2 times/week | 0.82 (0.41–1.66) | Age , sex, job status, years of  education, body mass index, time engaging in sports or exercise, time spent walking , cigarette smoking,  alcohol drinking, history of hypertension, diabetes mellitus and  gastric ulcer, family history of cancer, daily total energy intake, consumption of rice, daily consumption of miso soup, daily consumption of soybean products, total meat, total fish, dairy products, other fruits, total vegetables and consumption of oolong tea, black tea, coffee and green tea |
| George, 2009, USA | NIH-AARP Diet and Health Study | 1995/1996-2003, | 483338 participants (288109 M 195229 F), age 50-71y, 1664 cases (1406 M, 258 F) | Bladder cancer | Validated 124-item FFQ | Vegetables | 2.46 vs. 0.26  cup equivalent/1000kcal | 0.94(0.80-1.11) | Age, smoking, energy intake (log-transformed kcal), BMI, alcohol, physical activity, education, race, marital status, family history,  menopausal hormone therapy, and vegetable or fruit intake. |
|  |  |  |  |  |  | Fruits | 2.4 vs. 0.4  cup equivalent/1000kcal | 0.98(0.83-1.15) |  |
| Larsson, 2008,  Sweden | Swedish Mammography Cohort and Cohort of Swedish men | 1987/1994-2007, 9.7 years | 82002 participants (45338 M 36664 F), age 45-75y, 485cases (409M, 76F) | Bladder cancer | Validated 96-item FFQ | Fruits and vegetables | ≥5.8 vs. <2.7 servings/day | 0.80(0.60-1.05) | Age, sex, education (primary school, high school, and university), smoking status (never, past, and current), and pack-years of smoking (<20, 20-39, and >40 pack-years), and total energy intake. |
|  |  |  |  |  |  | Vegetables | ≥3.7 vs. <1.6 servings/day | 0.89(0.67-1.19) |  |
|  |  |  |  |  |  | Cruciferous vegetables | ≥3.5 vs. <0.9 servings/week | 0.97(0.74-1.27) |  |
|  |  |  |  |  |  | Leafy vegetables | ≥4 vs. <0.9 servings/week | 0.97(0.74-1.24) |  |
|  |  |  |  |  |  | Fruits | ≥2.3 vs. <0.8 servings/day | 0.93(0.69-1.25) |  |
|  |  |  |  |  |  | Citrus fruits | ≥5.1 vs. <0.5 servings/week | 0.88(0.68-1.16) |  |
| Iso, 2007,  Japan | Japan Collaborative Cohort Study (JACC Study) | 1988/1990- 2003, 15 years | 105500 participants (43011 M, 59504 F), age 40-79 y, 124 cases (85 M, 39 F) | Urothelial  cancer | Validated 39-item FFQ | Citrus fruits | ≥5.0 vs. <3.0 servings/week | 0.62(0.36-1.06) | Age, area of study. |
| Sakauchi, 2004, Japan | Japan Collaborative Cohort study for Evaluation of Cancer Risk | 1988/1990-1999,  9.8  years | 114517 participants (47997 M 66520 F), age 40-80 y, 88 cases  (63 M, 25 F) | Urothelial  cancer | Validated 32-item FFQ | Leafy vegetables | Almost every day vs. <=1-2 servings/week | 0.76(0.42-1.38) | Sex, age, and smoking index. |
| Holick, 2005,  USA | Nurses’ Health Study | 1976/1980-2000, 20 years | 88796 female participants, age 30-55y, 237 cases | Bladder cancer | Validated 130-item  FFQ | Fruits and vegetables | 6.8 vs. 1.9 servings/day | 1.08(0.70–1.65) | Age, pack-years of cigarette smoking, current smoking, and total caloric intake. |
|  |  |  |  |  |  | Vegetables | 3.7 vs. 1.1 servings/day | 1.29(0.87–1.91) |  |
|  |  |  |  |  |  | Cruciferous vegetables | >6.0 vs. 0.9 servings/week | 1.10(0.76–1.60) |  |
|  |  |  |  |  |  | Fruits | 3.8 vs. 0.6 servings/day | 0.95(0.62–1.46) |  |
|  |  |  |  |  |  | Citrus fruits | 14.0 vs. 0.9 servings/week | 0.96(0.66–1.42) |  |
| Michaud , 2002,  Finland | ATBC study | 1985/1988-1998, 11 years | 27111 male smokers participants, age 50-69y, 344 cases | Bladder cancer | Validated 276-item  FFQ | Fruits and vegetables | 422.3 vs. 83.6 g/day | 1.28(0.891.84) | Age, duration of smoking, smoking dose, total energy and trial interventions (alpha-tocopherol and beta-carotene supplements). |
|  |  |  |  |  |  | Vegetables | 205.3 vs. 39.5 g/day | 1.16(0.82-1.63) |  |
|  |  |  |  |  |  | Cruciferous vegetables | 33.0 vs. 0  g/day | 1.15(0.83-1.60) |  |
|  |  |  |  |  |  | Fruits | 245.4 vs. 25.0 g/day | 1.10(0.77-1.57) |  |
| Zeegers, 2001, Netherlands | Netherlands Cohort Study | 1986-1992, 6.3 years | 120852 participants (58279 M 62573 F), age 55-69y, 538 cases (463 M, 75 F) | Urothelial cancer | Validated 150-item  FFQ | Fruits and vegetables | ≥471 vs. <241g/day | 0.98(0.60-1.61) | Age, sex, number of cigarettes per day, years of cigarette smoking, total vegetable consumption (grams/day for fruit items), total fruit consumption (grams/day for vegetable items). |
|  |  |  |  |  |  | Vegetables | ≥242 vs. <126 g/day | 0.91(0.65-1.27) |  |
|  |  |  |  |  |  | Leafy vegetables | ≥33 vs. <9  g/day | 0.89(0.65-1.23) |  |
|  |  |  |  |  |  | Fruits | ≥256 vs. <83 g/day | 0.74(0.53-1.04) |  |
|  |  |  |  |  |  | Citrus fruits | ≥128 vs. <15 g/day | 0.85(0.62-1.17) |  |
| Nagano, 2000, Japan | Life Span Study, atomic bomb survivors, Japan | 1979/1981-1993 | 38540 participants (14873 M 23667F) age mean 55y, 114 cases (83 M, 31 F) | Bladder cancer | 22-item  FFQ | Fruits | >5 vs.0-1 servings/week | 0.75(0.46-1.22) | Age, gender, radiation dose, smoking status, education level, body mass index, calendar time, green-yellow vegetables and chicken consumption. |
| Michaud, 1999, USA | Health Professionals Follow-up Study | 1986-1996, 10 years | 51529 male participants , age 40-75y, 252 cases | Bladder cancer | Validated 131-item  FFQ | Fruits and vegetables | 9.9 vs. 2.7 servings/day | 0.75(0.49-1.14) | Age, pack-years of cigarette smoking, current smoking status, geographic region, total fluid intake and caloric intake. |
|  |  |  |  |  |  | Vegetables | 6.3 vs. 1.5 servings/day | 0.72(0.47-1.09) |  |
|  |  |  |  |  |  | Cruciferous vegetables | 7.0 vs. 0.5 servings/week | 0.49(0.32-0.75) |  |
|  |  |  |  |  |  | Leafy vegetables | 6.4 vs. 0 servings/week | 0.99(0.63-1.56) |  |
|  |  |  |  |  |  | Fruits | 4.5 vs. 0.6 servings/day | 1.12(0.70-1.78) |  |
| Chyou, 1993, Hawai | Honolulu Heart Program | 1965/1968-1991,  22 years | 7995 male participants, 46-65, 96 cases | Bladder cancer (lower urinary tract) | 17-item  FFQ and 24 hours diet recall | Fruits | ≥ 5vs. ≤1 servings/week | 0.63(0.37-1.08) | Age and smoking habit. |
| Shibata, 1992, USA | Laguna Hills Study USA | 1981/1985- 1989 | 11580 participants, age 65-84 y, 71 cases | Bladder cancer | FFQ | Fruits | 4.38 vs. 1.35 servings/day | 0.56(0.28-1.11) | Age and smoking. |
| Steineck, 1988, Sweden | Swedish Twin Cohort | 1969-1982, 14 years | 16477 participants, age 44-89y, 80 cases (63 M, 17 F) | Urothelial cancer | 8-item  Food questionnaire | Fruits and vegetables | Yes vs. No | 1.00(0.60-1.60) | Age and sex. |

M abbreviation for male

F abbreviation for female
