# Supplementary figures and images for: Fruits, vegetables, and bladder cancer risk: a systematic review and meta-analysis
Source: Cancer Med. 2014 Dec 2;4(1):136–46. doi: 10.1002/cam4.327 (PMC4312127; doi:10.1002/cam4.327)

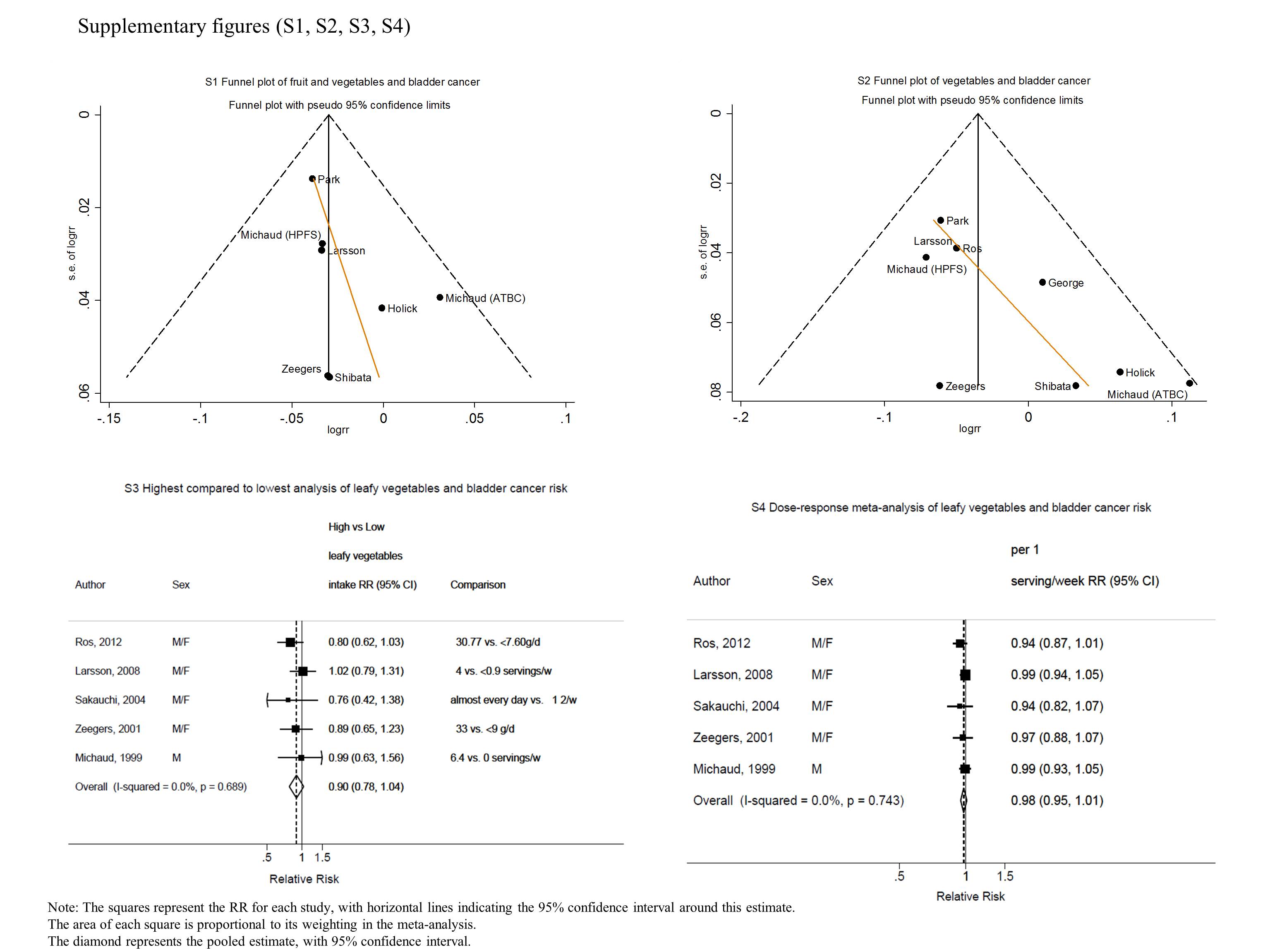

Supplement: Supplementary file 2 [file cam40004-0136-sd2.jpg]
